# Venetian Rule and Control of Plague Epidemics on the Ionian Islands during 17th and 18th Centuries

**DOI:** 10.3201/eid1501.071545

**Published:** 2009-01

**Authors:** Katerina Konstantinidou, Elpis Mantadakis, Matthew E. Falagas, Thalia Sardi, George Samonis

**Affiliations:** National and Kapodistrian University of Athens, Athens, Greece (K. Konstantinidou); University Hospital of Heraklion, Heraklion, Crete, Greece (E. Mantadakis, G. Samonis); Alfa Institute of Biomedical Sciences, Marousi, Greece (M.E. Falagas, T. Sardi)

**Keywords:** Plague, social and political measures, infections, Ottoman Empire, Europe, Ionian Islands, Greece, Venice, historical review

## Abstract

During the 17th and 18th centuries, measures were taken by the Venetian administration to combat plague on the Ionian Islands. At that time, although the scientific basis of plague was unknown, the Venetians recognized its infectious nature and successfully decreased its spread by implementing an information network. Additionally, by activating a system of inspection that involved establishing garrisons along the coasts, the Venetians were able to control all local movements in plague-infested areas, which were immediately isolated. In contrast, the neighboring coast of mainland Greece, which was under Ottoman rule, was a plague-endemic area during the same period. We conclude that even in the absence of scientific knowledge, close observation and social and political measures can effectively restrain infectious outbreaks to the point of disappearance.

## Abstract 2

One-sentence summary for tables of contents: Measures taken by the Venetian administration to combat plague were successful.

Plague is a zoonotic infection circulating among small animals, usually black rats and their fleas; it is caused by the bacillus *Yersinia pestis*. This disease is transmitted from animals to humans by the bite of infected fleas, direct contact, inhalation, and, rarely, ingestion of infective material. Untreated plague has a high case-fatality rate (*1*,*2*).

*Y*. *pestis* is a global pathogen that has active foci in all continents except Australia and Antarctica (*3*). Plague represents an exotic disease in North America; it usually affects prairie dogs (*Cynomus luduvicianus*) and has eliminated large colonies of these animals in the northwestern United States. Although these animals are susceptible, it is believed that other rodents and their fleas are the reservoirs and spread the disease during epizoonotics and maintain the pathogen (*4*).

Three forms of plague are known: bubonic, septicemic, and pneumonic. The bubonic form is most common and results from the bite of an infective flea. The bacillus enters through the bite, travels through the lymphatic system to the lymph nodes, and results in painful inflammation. The septicemic form occurs when the infection spreads through the bloodstream. The pneumonic form results from inhalation of aerosolized infective droplets and can also be transmitted between humans (*1*,*2*).

Bubonic plague, historically also known as Black Death, swept across Europe during the late medieval period in an epidemic that started in 1347 (*5*–*9*). The disease got its name from the deep purple, almost black discoloration of infected persons caused by subcutaneous hemorrhages. Wars, poverty, hunger, and malnutrition made Europe of the 14th century an ideal ground for plague epidemics. The first major outbreak occurred in Sicily in 1347, spread through Europe, and killed nearly half of the population (≈25 million persons) in 3 years. The disease became endemic and haunted the continent throughout the 14th–18th centuries. Major outbreaks occurred in Italy in 1629, London in 1665, and Vienna in 1679 (*5*–*9*).

It is not known where the pandemic started. It most likely originated in central Asia and was carried west by Mongols and traders along the Silk Road. It was imported to Europe through Crimea, from which it spread to Sicily. The total number of deaths worldwide from the pandemic is estimated to be 75 million (*5*–*9*).

Urban rat-borne plague has been controlled since the beginning of the 20th century by modern sanitation practices. Epidemics caused by rats transferred on ships to port cities are no longer a threat. However, the disease still occurs in rural areas because *Y*. *pestis* infects various wild rodents (*1*,*2*).

The continuing potential for reemergence of plague is evident by reports of outbreaks of the infection in Africa and India. The World Health Organization reports 1,000–3,000 new cases every year in impoverished rat-infested rural areas of Africa, Southeast Asia, and South America (*1*,*2*). However, an investigation has created some doubts about the nature of Black Death by implicating other possible causes such as Ebola-like viruses or other infectious agents (*10*).

Since 1347, when plague appeared in Europe, and especially after 1493, when syphilis was observed in Europe, theories on infectious and communicable diseases were formulated. Many scientists during these times developed ideas of contagion, which had an effect on public health regulations and the structure of cities. Moreover, decisions on plague control during that period reinforced the idea of public health measures for prevention of infectious diseases, an idea that was previously vague.

Since the 11th century, Venice, a naval and commercial power, had a special interest in the Eastern Mediterranean and later took advantage of the redistribution of the land of the Byzantine Empire after the Fourth Crusade (1204). Gradually and through conflicts after this crusade, this city-state on the Adriatic Sea gained control of the Ionian Islands, Crete, and some coastal cities of mainland Greece and established a network of trading posts. The islands of Corfu (Greek name Kerkyra), Zante (Zakynthos), Cephalonia, and Leukada were incorporated into the Venetian State in 1386, 1485, 1502, and 1684, respectively, and remained part of it until its demise in 1797 (*11*).

Our study investigates plague on the 4 islands during the 17th and 18th centuries. This period was selected because after the second half of the 17th century plague was observed only sporadically with limited epidemics. Plague was last observed in Venice in 1630, whereas in southeastern Europe, plague was observed until the 19th century (*12*). During the early 18th century, changes took place in the Venetian health policies, and the strategic and economic role of the islands increased after the gradual loss of the great trade routes of the Mediterranean Sea.

The 4 large islands in the Ionian Sea (Figures 1, 2) are a useful area for research because they had been under Venetian rule and were located just off the western coast of mainland Greece. This location made them a gateway to and from the Ottoman Empire and a frontier of Venice to the East. However, despite their proximity to mainland Greece, political and institutional differences were substantial between these islands and the neighboring Greek coast that was under Ottoman rule. Research regarding plague in this area has been limited. Moreover, comparisons of Venetian health policies regarding plague and their effectiveness on the Ionian Islands with those of neighboring Greece have not been made.

**Figure 1:**
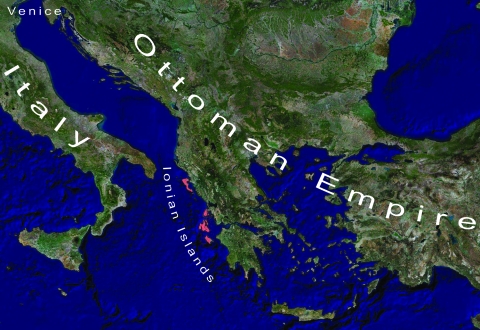
Map of the wider geographic area of reference.

**Figure 2:**
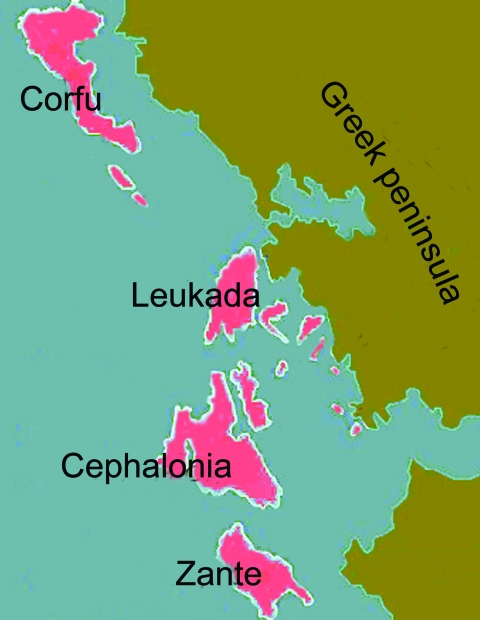
The 4 large Ionian Islands, which were under Venetian rule, and the Greek peninsula, which was under Ottoman rule, during the period studied (17th and 18th centuries).

Our study had 4 goals. The first goal was to identify epidemics of plague that struck the Ionian Islands during the 17th and 18th centuries. The second goal was to reconstruct the course of the epidemics. The third goal was to highlight differences in the prevalence of infection on the Ionian Islands during the 17th and 18th centuries and discuss the epidemiologic status of the islands compared with that of the neighboring coast of the Greek peninsula. The fourth goal was to investigate and describe measures taken by the Venetian authorities on these islands against plague during the study period.

## Historical Sources

On-site research was conducted in the Venetian state archive (Archivio di Stato di Venezia). Unpublished archival material dealing with the Ionian Islands during the 17th and 18th centuries was investigated with emphasis on periods of epidemiologic crises. We studied the following: 1) total number of registers of legislative bodies of the Venetian Republic (Senato mar and Senato rettori) for 1600–1797 to locate laws dealing with curtailment of plague and organization of health services on the Ionian Islands, 2) daily correspondence of Venetian authorities of the islands with Venice through the *proveditori da terra et da mar* and *senato* (secreta) *dispacci rettori*, and 3) archives of the Venetian health inspectors (*provveditori alla sanità*) regarding the Venetian health policy for the Ionian Islands. Additionally, several historical sources providing information about plague epidemics in the Mediterranean area during the 17th and 18th centuries were reviewed. This investigation was related to the doctoral dissertation of this article’s first author (K.K.), a historian at the University of Athens. This dissertation (*13*) reviews the subject historically. This article examines the subject from medical and epidemiologic points of view.

## Findings and Discussion

Archival sources show that most cases of plague on the Ionian Islands during the 17th and 18th centuries were imported from the neighboring coast of mainland Greece and ports in the southwestern Ottoman Empire. Only 2 epidemics were imported from the trade routes of the Mediterranean Sea. Of 11 epidemics, 8 occurred during the 17th century and 3 occurred during the 18th century. Plague struck Corfu in 1611, 1630, 1648, and 1673; Zante in 1617, 1646, 1692, and 1728; and Cephalonia in 1646 and 1760. Leukada had a disastrous epidemic in 1743, a few decades after the island became part of the Venetian Republic.

During the 18th century, plague had waned despite an outbreak along the southwestern coast of the Ottoman Empire, a short distance from the coasts of the Ionian Islands. In the 18th century, the southern Balkans had repeated waves of plague in 1718–1720, 1728–1731, 1733–1740, 1756–1765, 1782–1784, 1787–1789, and 1790–1793 (*13*). Only 14 plague-free years are described for the Greek peninsula during the 18th century (*14*). These epidemics affected cities and villages in western Peloponnese and western mainland Greece, which, because of trade, were in constant contact with the Ionian Islands. Because of commercial interests, contact between inhabitants of the islands and mainland Greece could not be halted. However, vigorous attempts by Venetian authorities stopped all communication between these areas during plague outbreaks.

Under these conditions, waning of plague epidemics during the 18th century may be attributed to sanitary measures taken by the Venetian government. These measures, among other regulations, dictated strict control of population movements, particularly during periods of epidemiologic crises. The efficacy of these measures is better appreciated because plague during the 17th century spread from *lazzarettos* (institutions where those with plague or other similar diseases were isolated) mainly because of negligence. In contrast, 2 of the 3 epidemics during the 18th century were caused by incorrect diagnoses or delayed notification of the authorities. Success of these measures became apparent in Corfu where plague was eradicated after 1673, only to reappear during British rule in the early 19th century (*13*). The importance of Corfu to Venice, particularly after the loss of Crete in 1669, resulted in creation of an effective invisible wall against plague until the end of the Venetian domination in 1797.

The city-states of northern Italy, including Venice, organized their defense against plague from the time of the Black Death (*9*). Experience and observation provided the first tools against epidemics because scientific information about the cause of plague was not obtained until several centuries later at the end of the 19th century, through laboratory research conducted by Alexandre Yersin (*15*). The Venetian State, on the basis of the belief in the miasmatic and contagious nature of plague and being a pioneer in organization of public health services conceived in the late Middle Ages, established regulations and practices in the city of the Doges and its conquests. These regulations and practices included quarantine (period of isolation ranging from 14 to >40 days and occasionally even longer, depending on the health of the port of origin), *lazarettos*, public health offices, and *cordoni di sanità*, which on the Ionian Islands were coastal garrisons that controlled access to Venetian territories (*16*).

Archival sources showed that health board officers in Corfu and Zante were initially elected around 1545. However, it is likely that health boards had previously existed. The first *lazzarettos* were established in Corfu and Zante in 1588 and in Cephalonia and Leukada at the beginning of the 18th century.

The Venetian health policy was reformed in the 18th century. These reforms likely emphasized disease prevention and dealt with health emergencies (*17*). The new policy was based on daily reports of health conditions on the islands and suspicious areas in the eastern Mediterranean, and included creation of a common public health framework (identical laws for plague control, decrees, and institutions and infrastructures) for all islands. This policy would facilitate interventions and change the way in which local health officers were selected.

The main goal of these reforms was improvement of the structure and function of *lazzarettos*. During the 18th century, new *lazzarettos* were established, and existing ones underwent extension and changes. In 1726, a new statute defining the obligations and responsibilities of the heads of the institutions was introduced. According to this statute, during periods with no or low disease activity, *lazzarettos* referred directly to Venetian authorities and bypassed authority of local health boards (*13*).

Despite these new regulations, Venetian archives reported occasional problems in health services of the islands because of poorly trained health officers, who were elected from the local upper class and were eager to acquire greater political autonomy. This situation resulted in conflicts between Venetian representatives and local health officials, as well as between local factions known as *cittadini* (*18*).

Archives show that until the end of the Venetian rule on the Ionian Islands, *lazzarettos* functioned as protective shields for Venice and transferred responsibility of plague control from Venice to peripheral areas (*19*). When there was evidence or even suspicion that plague was present on an island, all links to Venice were immediately discontinued for the duration of the threat. Islands were isolated by order of the *senato*, and trade commenced only after the state of emergency had ended. During these periods, the Venetian government did not intervene in the responsibilities and actions of local health inspectors because such intervention could provoke social unrest (*13*). Thus, the cost for Venice was minor. However, this action did not indicate negligence by the state mechanism each time plague affected an island and threatened Venice.

Prevention was based on widespread use of an information network of daily reports of Venetian consuls in Mediterranean areas to Venetian authorities, detailed interrogation of sailors who arrived in Venetian ports, effective control of all local movements in plague-infested areas, and activation of the *cordoni di sanità*. Additionally, when plague occurred, residents were separated by health authorities into groups of healthy and sick persons regardless of social hierarchies. Persons affected by plague were kept in *lazarettos*, and large numbers of infected persons were kept in hospitals, houses, or neighborhoods on the assumption that plague in these persons remained isolated. Isolation was ensured by military force. Thus, plague-stricken areas resembled a large institution under constant inspection and surveillance and disconnected from the rest of society (*13*).

In contrast to measures taken on the Ionian Islands during the 17th and 18th centuries, mainland Greece, which was under Ottoman rule, had a different mentality in dealing with plague. Isolation of patients and quarantine were not common practices. Thus, plague continued to cause epidemics in mainland Greece during the first half of the 19th century. These epidemics had devastating demographic and financial consequences. After 1830, when the Ottoman administration implemented sanitary measures such as quarantine, spread of plague in mainland Greece decreased substantially (*20*).

Although modern medical regimens can successfully treat patients with plague and stop its spread, prevention and isolation policies can contribute to control of this disease. This finding has been demonstrated by successful management of a plague epidemic in Surat, India, in 1994, in which prevention techniques similar to those used during Venetian rule were used (*21*)

In conclusion, although the scientific basis of plague was not known, the Venetian administration recognized the infectious nature of this disease and took successful measures that dramatically decreased the spread of the plague epidemic on the Ionian Islands during the 18th century. The results of these measures are more impressive if compared with those in the neighboring coastal region of the Greek peninsula, which under Ottoman rule had endemic plague during the same period. Results of the present historical investigation lead to the conclusion that even in the absence of scientific knowledge, observation and well-organized public health services can effectively restrain infectious outbreaks to the point of disappearance, as occurred with plague in Corfu during the 18th century.
